# The feasibility and effectiveness of remote cognitive training on cognitive function and work performance in workers

**DOI:** 10.3389/fpsyg.2024.1404518

**Published:** 2024-07-31

**Authors:** Michi Shibaoka, Masashi Masuda, Satoko Iwasawa, Satoru Ikezawa, Hisashi Eguchi, Kazuyuki Nakagome

**Affiliations:** ^1^Yokohama Rosai Hospital, Japan Organization of Occupational Health and Safety, Kanagawa, Japan; ^2^Smart OHW Co., Ltd., Tokyo, Japan; ^3^Preventive Medicine and Public Health, National Defense Medical College, Saitama, Japan; ^4^National Center of Neurology and Psychiatry, Tokyo, Japan; ^5^Department of Mental Health, Institute of Industrial Ecological Sciences, University of Occupational and Environmental Health, Fukuoka, Japan

**Keywords:** mental health, cognitive function, work performance, occupational health, psychosocial health

## Abstract

**Objectives:**

We aimed to determine whether remote cognitive training (CT) is feasible and has the potential to improve cognitive function and work performance in Japanese workers.

**Methods:**

From June to September 2020, this intervention time series study enrolled workers aged 18–65 years from 10 companies located in a metropolitan area of Japan. Cognitive function tests and self-administered questionnaires were completed by the participants three times: at baseline, after 12 weeks of CT, and after a further 12 weeks following cessation. We measured work performance with the question: “How would you rate your performance (compared with your optimum performance) over the past 4 weeks?” Responses were made via a visual analog scale (0–100). Cognitive function was assessed using the THINC-integrated tool, which is a brief, objective computerized cognitive assessment battery. For our computerized remote CT intervention, BrainHQ was used on the basis of our scientific rationale and the empirical literature. We recommended three 20-min sessions of BrainHQ per week and sent participants three reminders.

**Results:**

In total, 119 participants were recruited to this study. Only 22.7% of the subjects achieved the recommended training time of 720 min over 12 weeks. The median training time was used to divide participants into long and short- training groups. The long-training group showed a greater improvement in attention and executive function than the short-training group but there was no significant improvement in work performance after CT compared to baseline.

**Conclusion:**

Our results suggest that although remote CT was not feasible enough, the effects on cognitive function can be expected by increasing training time and motivation.

## 1 Introduction

Mental health in the workplace has emerged as a major issue due to the increasing prevalence of mental health problems. Over the past several decades, the economic costs associated with mental health disorders have increased and workplace productivity has decreased (Chisholm et al., 2016). According to the World Health Organization (WHO), mental health indicates “a state of well-being in which individuals are aware of their own abilities, can cope with the normal stresses of life, can work productively and fruitfully, and can contribute to their communities” (World Health Organization, 2004). As such, mental health is multifaceted and is viewed under a variety of definitions. Some define mental health as a continuum of cognitive functions related to thoughts, mood and emotions and behavior related to negative and positive mental health states (Montano et al., 2017). According to this definition, cognitive functions play an important role in regulating thoughts, feelings, and behaviors that lead to healthy mental health.

Naturally, cognitive impairment has emerged as a public health issue that has been shown to have a significant impact on occupational and daily functioning and quality of life in a wide range of health areas, such as the aging population (Wilson et al., 2013), mental disorders (Green et al., 2000), developmental disorders (Barkley, 1997; Romero-Ayuso et al., 2021), neurological diseases (Rao et al., 1991) and even cancer (Janelsins et al., 2014). The impact of cognitive function on mental health is seen as a population issue rather than in relation to specific diseases. It seems important to pay attention to the cognitive function of workers in order to improve mental health in the workplace and the performance associated with it. In our previous study, we found that subjective work performance was positively correlated with cognitive function in the general workforce. It was suggested that enhancing workers’ cognitive function could improve their work performance (Shibaoka et al., 2023).

Cognitive training (CT) has been shown to be effective against cognitive dysfunction in patients with various neuropsychiatric disorders (Cicerone et al., 2011; Wykes et al., 2011). It is usually carried out under the supervision of a therapist several times a week for several months, but due to the impact of the COVID infection disaster, it is increasingly being carried out remotely using ICT. While remote access has significant advantages for those who have distance and time difficulties in attending a therapist, it also has disadvantages in that they do not receive prompting from the therapist, and a study of cancer patients have shown that home-based intervention has not always resulted in good adherence (Engan et al., 2023). On the other hand, it is not clear how long or how much time is needed to show efficacy on cognitive function, so feasibility needs to be considered depending on the subject. In a trial of remote CT for older people, an intervention of only 3 min a day for 6 weeks showed significant improvements in cognitive function (Corbett et al., 2024).

It is expected that the general workforce may find it difficult to attend a therapist for CT due to their regular work, while they may be less motivated to engage in CT than older people or those with illnesses without the prompting of a therapist. Furthermore, as this is the first study to explore the potential of remote CT for the general workforce, it is difficult to determine the time and duration of training, however, with reference to previous studies we aimed to assess the feasibility of training adherence in the general workforce. Effectiveness, on the other hand, was assessed using a time series design analysis. Therefore, the results should be considered exploratory as there was no true control group in our study.

The importance of mental health is being increasingly recognized in occupational health, but there have been few attempts to integrate mental health care practice into occupational health, presumably because many companies feel unsure about what they can do to improve mental health. This study aimed to investigate whether remote CT, as a mental health care practice, can improve cognitive function and whether the change in cognitive function is associated with the change in work performance in Japanese workers as a new approach to mental health.

## 2 Materials and methods

### 2.1 Participants

The participants were Japanese workers recruited from 10 companies located in a metropolitan area of Japan between February and November 2019. All participants were aged 18–65 years. Prior to the CT, a cognitive function test and a self-administered online questionnaire were completed. We created a BrainHQ (Posit Sciences, San Francisco, CA, USA) account using each participant’s last name and ID and emailed the URL for the CT to the participants. We also emailed participants their IDs and login passwords.

We sent reminders to the participants regarding the training three times a week. We had authority as BrainHQ account administrators to monitor the training times of the participants. We recommended participants to train using their own smartphones, tablets, computers, or any other device allowing continuous training, approximately three sessions per week, 20 min per session. Participants completed cognitive function tests at baseline, after 12 weeks of CT (post-treatment), and 12 weeks after the cessation of CT (follow-up).

We explained the study in person, and those who showed an interest were invited to take part in CT. Written informed consent was obtained from all participants. The study protocol was approved by the Institutional Review Board of the Japan Organization of Occupational Health and Safety.

### 2.2 Measures

#### 2.2.1 Outcome measure

The primary outcome measure in this study was feasibility, which was assessed by the proportion of subjects who adhered to the recommended 720 min of training over 12 weeks.

Another outcome measure was change in cognitive function, which was assessed using the THINC-Integrated Tool (THINC-it^®^) (McIntyre et al., 2017). This tool was used because it is simple and easy to administer. THINC-it^®^ is a brief computerized battery of cognitive function tests available for use on personal computers and touchscreen tablet devices. The tests measure cognitive performance in multiple domains, and a subjective evaluation of cognitive function is conducted separately (McIntyre et al., 2017). THINC-it^®^ has been validated and widely used for assessing cognitive function in patients with mood disorders (McIntyre et al., 2020). According to a previous study with healthy volunteers, the psychometric data are quite good and acceptable. Test–retest reliability varied between 0.75 and 0.8, intrarater reliability between 0.7 and 0.93, and test stability, calculated by the within-subject deviation value for each of the THINC-it measures, yielded values between 5.9 and 11.23 for accuracy measures and 0.735 and 17.3 s for latency measures, which were considered acceptable. Convergent validity, using correlation with other comparison tasks, was in the acceptable range for three tasks, but was low for the Symbol Check task (Harrison et al., 2018). Moreover, THINC-it^®^ has been translated into multiple languages, including Japanese, and can be downloaded for free. Completing all THINC-it^®^ components takes 10–20 min, and the task instructions are designed to minimize administrative requirements. We considered 10–20 min acceptable for a survey conducted in the workplace. The tests were administered in the following order: the self-reported Perceived Deficits Questionnaire – Depression-5 item (PDQ-5-D), Spotter, Symbol Check, Codebreaker, and Trails. Of these five tasks, the last four test objective cognitive function (attention/concentration, executive function, working memory, processing speed). A validation study of THINC-it® demonstrated that subjective and objective cognitive function were impaired in patients with depression relative to healthy controls (McIntyre et al., 2020). Under the guidance of a psychiatrist, a psychologist, and occupational physicians, participants completed the THINC-it^®^ cognitive function tests using a 9.7-inch tablet computer.

The five questions of the PDQ-5-D assess attention, planning, organization, and concentration during the previous 7 days. Participants rate the difficulty experienced in each domain on a Likert scale ranging from 1 (*Never*) to 5 (*Very often*). Higher PDQ-5-D scores indicate greater subjective cognitive impairment.

Spotter is a reaction time test of attention and executive function inspired by the choice reaction time task. Participants are presented with a left- or right-pointing arrow and are required to select the left or right direction as quickly as possible depending on the direction of the arrow. The latency to cue presentation varies among trials and the cue may appear on the left or right side of the screen; this can cause interference effect. The test comprises 40 trials and takes 2 min to complete. Participants are assessed according to their mean correct reaction time. In this study, responses made before 100 ms were treated as erroneous (anticipatory) responses.

Symbol Check evaluates working memory, executive function, and attention/concentration. Participants are presented with a continuously moving sequence of symbols, equivalent to an n-back task. As the sequence moves to the left of the screen, the symbols are hidden in a specific order. Participants are required to recall each hidden symbol as quickly as possible and press one of the five symbols presented at the bottom of the screen accordingly. The test consists of 40 trials and takes 2 min to complete. The number of correct responses is the outcome measure.

Codebreaker requires participants to match a list of symbols to corresponding numbers based on the legend. This task was inspired by the Digit Symbol Substitution Test and can identify deficits in the domains of executive function, processing speed, and attention/concentration. This test also takes 2 min to complete. A legend comprising numbers ranging from 1 to 6 and corresponding symbols is provided at the top of the screen. The number of correct symbols matched within 2 min is considered to represent cognitive performance.

Trails, inspired by the Trail Making Test (TMT), evaluates executive function, and comprises 18 connecting points. Participants must trace a line between letters and numbers alternatively, beginning with the letter “A” and proceeding to number “1” as quickly as possible; they continue until all letters and numbers have been connected. If the line touches a letter or number that is not the next one in the sequence, participants must restart from the last correct digit. A shorter completion time represents better cognitive performance.

The third outcome measure was self-reported work performance, which was evaluated using a single question (with responses made using a visual analog scale; range: 0–100): “How would you rate your performance (compared with your optimum performance) over the past 4 weeks?” We aimed to measure presenteeism regardless of the presence or absence of illness. Using the Stanford Presenteeism Scale as a guide, (Turpin et al., 2004) four psychiatrists, two industrial physicians, and a clinical psychologist created questions via which workers could self-assess their labor productivity, regardless of illness status.

#### 2.2.2 Exposure variable: cognitive training

We used BrainHQ for CT based on our scientific rationale and the empirical literature. It allows the researcher to verify the training time and has been employed in various previous studies. Many studies of mental illness such as depression and schizophrenia, and of older adults, have been carried out using BrainHQ (Hagen et al., 2020; Nuechterlein et al., 2022). BrainHQ modules focus on improving the basic neurocognitive processes of auditory discrimination, processing speed, working memory, verbal memory, and verbal reasoning. Our participants were free to choose the training options that were of most interest to them. The training time was confirmed to correspond to the actual time of operation by using the application’s administrator privileges. We divided the subjects into binomial subgroups with respect to training time at the central value and compared the two subgroups to reduce the practice effect in terms of efficacy on cognitive function as assessed by THINC-it^®^. We also compared the change in work performance between the long and short training groups to verify the effect of CT.

#### 2.2.3 Potential explanatory variables for the change in work performance

In order to explore factors related to changes in work ability, a multiple regression analysis was conducted using age, sex, depression tendency, intrinsic motivation, and cognitive training time (long and short) as explanatory variables in addition to changes in cognitive function, with changes in work performance as the dependent variable.

##### 2.2.3.1 Intrinsic motivation

Recent evidence indicates the importance of intermediate factors that link cognitive function and social functioning, such as social cognition and motivation (Gard et al., 2009). Motivation is generally subdivided into intrinsic and extrinsic ones. Ryan and Deci state that the intrinsic motivation is “the inherent tendency to seek out novelty and challenge, to explore and investigate, and to stretch and extend one’s capacities” (Ryan and Deci, 2000). On the other hand, extrinsic motivation is affected by external control, such as acquisition of reward or avoidance of punishment. Over 30 years of research intrinsic motivation has been suggested to be more closely associated with better performance, persistence and well-being compared to extrinsic motivation (Ryan and Deci, 2000). Consistent with their suggestion, Saperstein et al. (2011) demonstrated that intrinsic motivation was significantly related with work functioning in patients with schizophrenia (Choi et al., 2010).

We used the Intrinsic Motivation Inventory (IMI) to measure the participants’ intrinsic motivation for CT and to test its association with change in cognitive function and social functioning (Choi et al., 2010).

The IMI consists of 21 items distributed among three subscales (Interest/Enjoyment, Perceived Choice, and Value/Usefulness; 7 items for each subscale) and uses a Likert scale ranging from 1 (*Not at all true*) to 7 (*Very true*). The IMI subscale scores are summed to derive the total score, with higher scores indicating stronger trends in each domain.

##### 2.2.3.2 Depression

Because one of the core features of depression is its functional disability including poor work performance (McIntyre et al., 2020), we included depression tendencies as a potential explanatory variable for change in work performance. Depression tendencies were assessed using the Kessler Psychological Distress Scale (K6), which is a screening questionnaire for depressive symptoms (Kessler et al., 2002). The Kessler Psychological Distress Scale comprises six questions, with responses made using a Likert scale ranging from 0 (*Never*) to 4 (*Very often*). Participants scoring ≥5 points are considered to have a tendency toward depression (Sakurai et al., 2011).

### 2.3 Statistical analyses

We used SPSS software (version 26.0; IBM Corp., Armonk, NY, USA) for the analysis. Cases with missing or extreme values were excluded.

Participants were divided into long- and short- (median split) training time groups, and treatment efficacy was assessed by comparing the results between the two groups. In addition, demographic and other clinical characteristics were compared between groups to examine potential confounders, which may affect the results. If a potential confounder is identified, additional analyses are performed to adjust for the effect of the confounders. First, paired *t*-tests were performed to compare changes in cognitive function between baseline and the post-treatment (12 weeks) and follow-up (24 weeks) timepoints for each training time group. Next, we compared the changes in cognitive function between the baseline and the post-treatment and follow-up between groups using Student’s *t*-tests.

As stated above, multiple regression analysis was performed with change in work performance as the dependent variable and age, sex, depression tendency, intrinsic motivation, training time of CT and the change in cognitive function as explanatory variables.

We also tested the relationships between the efficacy of CT on cognitive function and intrinsic motivation, using Spearman’s rank correlation analysis to verify previous findings suggesting that intrinsic motivation mediates between cognitive function and social functioning in patients with schizophrenia (Nakagami et al., 2008; Gard et al., 2009; Saperstein et al., 2011; Green et al., 2012).

*P*-values <0.05 were considered statistically significant.

## 3 Results

**Figure 1:**
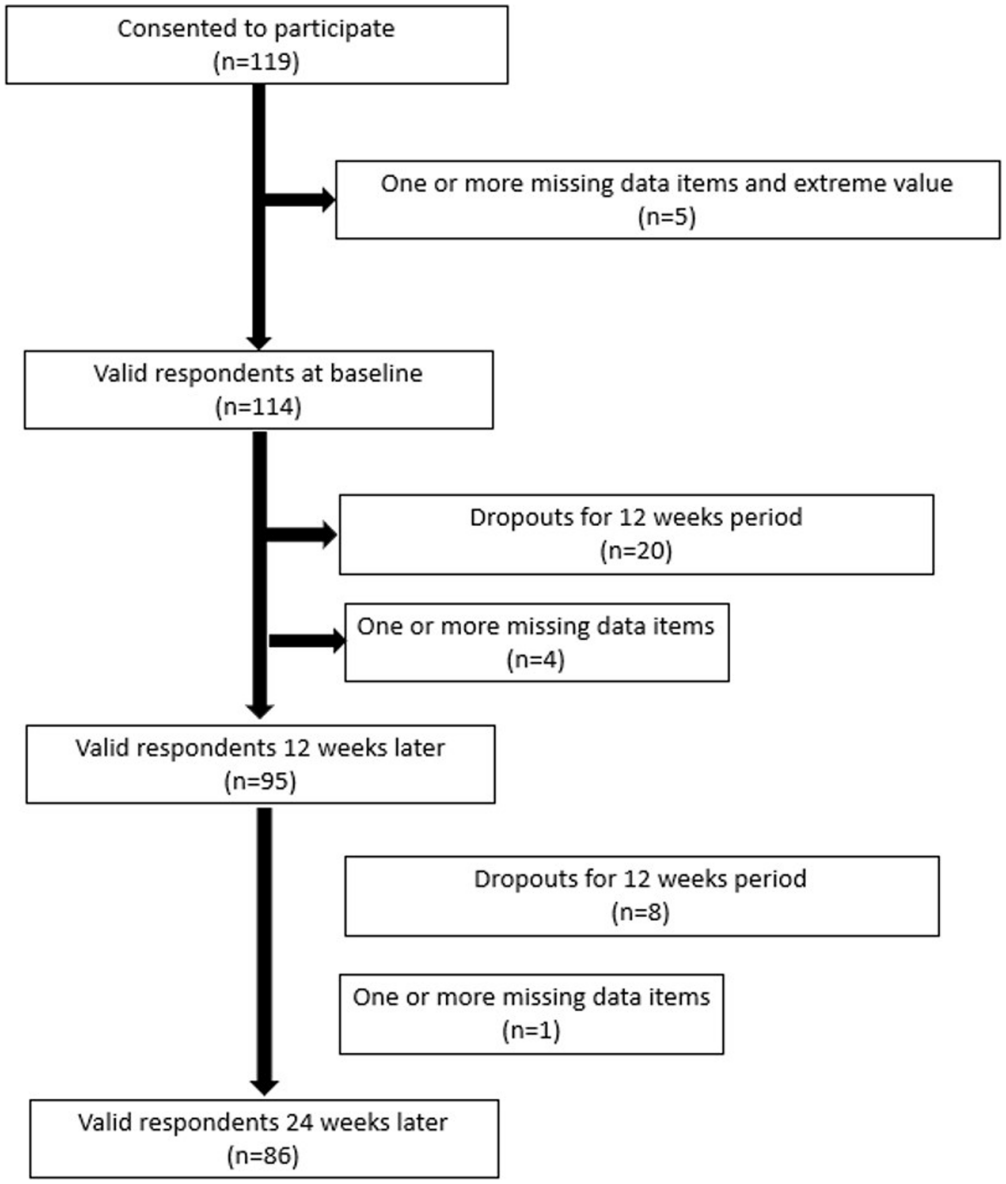
Flow of study participants based on the study exclusion criteria.

In total, 119 individuals provided written consent to participate in this study. A flow diagram of the study is shown in Figure 1.

The participants’ demographic and clinical characteristics are shown in Table 1. Most participants were full-time male workers.

**Table 1:** Sociodemographic characteristics (*n* = 119).

		*n*	%	Mean	SD
Age (years)		119		47.3	9.80
Sex	Male	76	63.9		
	Female	43	36.1		
	Other	0	0.0		
Employment status					
	Full-time worker	93	78.2		
	Part-time worker	21	17.6		
Employment position					
	Manager	31	27.0		
	Non-manager				
	Analyst/specialist	19	16.5		
	Sales representative	2	1.7		
	Technician/engineer	24	20.9		
	Administrative assistant	29	25.2		
	Others	10	8.7		
Sleep difficulties (AIS)					
	≥6	48	40.3		
	≤5	69	58.0		
Physical disease treatment status					
	Under treatment	22	18.5		
	Not treated	95	79.8		
Mental illness treatment status					
	Under treatment	3	2.5		
	Not treated	114	95.8		
Depression tendency (K6)					
	≥5	41	34.5		
	≤4	76	63.9		
ADHD tendency					
(ASRS v.1.1 part A)	+	14	11.8		
	−	100	84.0		
Autistic traits (AQ-J)					
	≥7	8	6.7		
	≤6	106	89.1		
Intrinsic motivation (IMI)		99			
	Interest/enjoyment			25.6	6.52
	Perceived choice			34.3	7.37
	Value/Usefulness			30.2	9.89
	Total			90.1	20.12

SD, standard deviation; AIS, Athens Insomnia Scale; K6, Kessler Psychological Distress Scale; ASRS, Adult ADHD Self-Report Scale v.1.1 part A; AQ-J, Autism-Spectrum Quotient-Japanese version; IMI, Intrinsic Motivation Inventory.

The proportion of the subjects who achieved 720 min of training time over 12 weeks was 22.7% (*n* = 27). Eleven subjects had no access to CT at all and the maximum training time was approximately 3,000 min, or 50 h. The mean (SD) value was 428.1 (594.84) minutes and the median was 125 min.

The participants with a training time above the median were classified into the long-training group. The long-training group showed higher intrinsic motivation for CT than the short-training group. The mean (SD) values of the long- and short-training times were 793.6 (655.41) minutes (*n* = 60) and 56.4 (64.18) minutes (*n* = 59), respectively.

Paired *t*-tests revealed a significant reduction in response time at follow-up compared to baseline in the short-training time group and at the post-treatment and follow-up in the long-training group in Spotter. Similarly, as for the other indices, there was a general tendency for improvement at the post-treatment and follow-up timepoints compared to baseline regardless of training time. Only for the Spotter, the long-training group showed a greater change between the post-treatment and baseline timepoints compared to the short-training group (56.4 msec vs. −4.2 msec). ANCOVA with the possible confounder IMI total score, which showed a significant difference between the long- and short-training time groups as a covariate, showed that the significant difference remained (*p* = 0.013). Regarding the change in work performance between the post-treatment, follow-up and baseline, although there was a significant decline at the post-treatment in the long-training group, no significant differences were found between the long- and short-training time groups (Table 2).

**Table 2:** Comparison between long- and short-training groups (*n* = 119).

				Long				Short		Long vs short
		*n*	%	Mean	SD	*n*	%	Mean	SD	*p* value
Age (years)		60		48.9	8.47	59		45.7	10.89	0.07^a^
Sex	Male	34	56.7			42	71.2			0.10^b^
	Female	26	43.3			17	28.8			
	Other	0	0			0	0			
Employment status										
	Full-time worker	50	83.3			43	72.9			0.37^b^
	Part-time worker	9	15.0			12	20.3			
Employment position										0.72^b^
	Manager	16	27.1			15	26.8			
	Non-manager									
	Analyst/specialist	11	18.6			8	14.3			
	Sales representative	2	3.4			0	0.0			
	Technician/engineer	12	20.3			12	21.4			
	Administrative assistant	14	23.7			15	26.8			
	Others	4	6.8			6	10.7			
Sleep difficulties (AIS)										
	≥6	27	45.0			21	35.6			0.37^b^
	≤5	33	55.0			36	61.0			
Physical disease treatment status										
	Under treatment	13	21.7			9	15.3			0.37^b^
	Not treated	46	76.7			49	83.1			
Mental illness treatment status										
	Under treatment	1	1.7			2	3.4			0.55^b^
	Not treated	58	96.7			56	94.9			
Depression tendency (K6)										
	≥5	22	36.7			19	32.2			0.71^b^
	≤4	38	63.3			38	64.4			
ADHD tendency										
(ASRS v.1.1 part A)	+	8	13.3			6	10.2			0.67^b^
	−	51	85.0			49	83.1			
Autistic traits (AQ-J)										
	≥7	5	8.3			3	5.1			0.53^b^
	≤6	54	90.0			52	88.1			
Intrinsic Motivation (IMI)		57				42				
	Interest/enjoyment			26.8	6.23			23.9	6.61	0.03^a^
	Perceived choice			35.3	7.01			33.0	7.71	0.12^a^
	Value/usefulness			32.0	9.19			27.8	10.28	0.04^a^
	Total			94.1	18.58			84.7	21.07	0.02^a^

SD, standard deviation; AIS, Athens Insomnia Scale; K6, Kessler Psychological Distress Scale; ASRS, Adult ADHD Self-Report Scale v.1.1 part A; AQ-J, Autism-Spectrum Quotient-Japanese version; IMI, Intrinsic Motivation Inventory.

^a^ Student’s *t*-test.

^b^ Pearson’s chi-square test.

The results of the multiple regression analysis are shown in Table 3. Neither the training time of CT nor the change between post-treatment and baseline in Spotter was not associated with the change in work performance. On the other hand, IMI total score showed a positive and the post-treatment to baseline change in Symbol Check showed a negative relationship with improvement in post-treatment work performance compared to baseline. Improvement in work performance was associated with higher intrinsic motivation for CT, but also with a worsening of Symbol Check scores.

**Table 3:** Longitudinal change in cognitive function/work performance and comparison of changes between long and short training time groups.

		Baseline		12 weeks later		24 weeks later		
	*n*	Mean^1^	SD^2^	Mean^1^	SD^2^	Mean^1^	SD^2^	*p* value
Spotter (msec)								
Short group	42	465.9	1.22	470.1	1.23			0.73
	37	469.1	1.23			447.6	1.21	<0.05
Long group	53	519.1	1.25	462.7	1.23			<0.05
	50	515.7	1.24			477.1	1.22	<0.05
*p* value	^a^	0.05		0.73		0.15		
	^b^			<0.05		0.38		
Symbol check (number)								
Short group	41	28.8	1.74	30.6	1.58			0.44
	36	29.1	1.74			34.2	1.32	<0.05
Long group	53	31.1	1.51	33.5	1.32			0.17
	50	31.0	1.53			34.3	1.38	0.08
*p* value	^a^	0.93		0.44		1.00		
	^b^			0.67		0.57		
Codebreaker (number)								
Short group	42	61.8	13.4	69.1	12.2			<0.05
	37	60.3	12.9			72.5	13.3	<0.05
Long group	53	64.4	10.6	65.5	17.2			0.69
	50	64.8	10.6			69.2	14.0	0.08
*p* value	^a^	0.61		0.24		0.15		
	^b^			0.11		0.06		
Trails (sec)								
Short group	42	22.5	1.48	20.1	1.45			<0.05
	37	23.1	1.47			20.7	1.43	0.06
Long group	53	23.0	1.38	21.3	1.41			0.05
	50	23.1	1.38			20.5	1.39	<0.05
*p* value	^a^	0.31		0.49		0.91		
	^b^			0.88		0.52		
PDQ-5D (point)								
Short group	42	4.0	1.90	4.2	1.87			0.53
	37	4.2	1.86			4.0	1.99	0.63
Long group	53	4.3	1.91	3.9	1.92			<0.05
	50	4.4	1.91			3.9	2.00	0.15
*p* value	^a^	0.34		0.77		1.00		
	^b^			0.14		0.51		
Work performance								
Short group	42	78.5	15.12	76.5	17.96			0.43
	36	78.2	15.68			78.8	17.78	0.25
Long group	56	84.3	14.06	78.8	12.43			<0.05
	50	84.4	14.70			80.9	14.27	0.08
*p* value	^a^	<0.05		0.49		0.54		
	^b^			0.27		0.25		

^1^Geometric mean (Spotter, Symbol check, Trails, and PDQ) and arithmetic mean (Code breaker).

^2^Geometric standard deviation (Spotter, Symbol check, Trails, and PDQ) and standard deviation (Code breaker).

Spotter (Choice Reaction Time) = mean latency for correct response.

Symbol check (N-back) = total number of correct responses.

Codebreaker (Digit Symbol Substitution Test) = total number of correct responses.

Trails (Trail Making Test) = total time taken for completion.

PDQ-5D (Perceived Deficits Questionnaire for Depression-5 item) = total score (points).

^a^*t*-test for cross-sectional mean level.

^b^*t*-test for the change from the baseline value.

Next, to explore the possibility of intrinsic motivation as an intervening factor linking cognitive function and work performance, Spearman’s rank correlation analysis was performed to test the association between post-treatment and baseline changes in Spotter and IMI total scores. The results of Spearman’s rank correlation analysis showed a significant association with Rho = −0.26 (*p* = 0.013), indicating that IMI is a factor associated with both cognitive function and work performance changes (Table 4).

**Table 4:** Factors predicting change in work performance.

	Multiple regression model	
	Model 1^1^	Model 2^2^
Age	−0.11	0.03
Sex	−0.01	0.03
Depression tendency (K6)	<0.01	−0.01
IMI total	0.21^*^	0.32^*^
Training time (long, short)	−0.11	−0.12
Spotter	0.11	0.20
Symbol check	−0.13	−0.25^*^
Codebreaker	0.20	0.14
Trails	−0.28^*^	−0.17
PDQ-5D	−0.06	−0.21

^*^p < 0.05.

^1^Unajusted multiple regression model.

^2^Fully adjusted logistic regression model: adjusted for sex, age, tendency for depression, intrinsic motivation, training time for cognitive training, and change of cognitive function.

Spotter (Choice Reaction Time) = the change of mean latency for correct response from baseline to 12 weeks later.

Symbol check (N-back) = the change of total number of correct responses.

Codebreaker (Digit Symbol Substitution Test) = the change of total number of correct responses.

Trails (Trail Making Test) = the change of total time taken for completion.

PDQ-5D (Perceived Deficits Questionnaire for Depression-5 item) = the change of total score (points).

## 4 Discussion

To our knowledge, this is the first study to investigate the feasibility and effectiveness of remote CT on cognitive function and its relationship with secondary work performance in Japanese workers.

First, feasibility, as measured by the proportion of subjects adhering to the recommended 720 min of training time, was inadequate. Only 22.7% of the subjects met the recommended total training time, which was much lower than the adherence rate in a study of cancer patients, which also showed a suboptimal rate of 65.5% in an unprompted home-based intervention (Engan et al., 2023). In another study of cancer patients (Bray et al., 2017), it was reported that the average total training time was 25.06 h of the recommended 40 h and only 27% of the patients completed the program within the recommended timeframe. The low adherence rate may be due to a lack of prompting from the therapist and therefore a lack of motivation to train themselves. In fact, the present study showed that intrinsic motivation for CT was associated with training time and also with changes in both cognitive function and work performance. To improve feasibility and effectiveness, subjects must be made fully aware that improving cognitive function through cognitive training is useful for improving work performance.

Although the results on effectiveness were highly exploratory due to the lack of an RCT and the use of a before and after comparison, by dividing the subjects into long- and short-training time groups, an improvement in attention and executive function was indicated by a greater change in Spotter in the long-training group than in the short-training group. In addition, according to a fully adjusted multiple regression analysis, higher IMI total scores and decreased test scores in Symbol Check contributed to improvements in work performance. However, there was no improvement in work performance at post-treatment and follow-up, compared to baseline. Also, there was no association between training time or change in other cognitive functions and change in work performance.

In this study, we divided the subjects into two groups with respect to the median level of training time. Although before and after comparisons of cognitive function tests show significant improvements at post-treatment and follow-up compared to baseline at some points, we must be aware of the practice effect of the tests, which may falsely inflate the effect. By comparing the effect between long- and short-training groups it may cancel out the practice effect. Since previous studies suggest that longer treatment time and higher treatment intensity lead to better results (Medalia and Richardson, 2005; McGurk et al., 2007), we assumed that CT could be considered effective if the long-training group showed a greater change at post-treatment compared to baseline than the short-training group. There is no golden standard for treatment time and intensity, but Wykes et al. suggest that the average length of treatment in the studies included in their meta-analysis for schizophrenia that confirmed robust efficacy was 32.2 h (range = 4–130), provided across 16.7 weeks (range = 2–104) and treatment intensity being 2.2 sessions per week (range = 0.6–5) (Wykes et al., 2011). In the present study, the average length of CT, even in the long-training group was around 13 h, which is far short compared to the above studies for schizophrenia. On the other hand, in studies of remote CT for older adults, an intervention as short as ten 60- to 75-min sessions (Ball et al., 2002), or even 3 min a day for 6 weeks showed significant improvements in cognitive function (Corbett et al., 2024). Although it is possible that the relatively short training time in the present study resulted in the limited efficacy, we do not yet know the appropriate duration and intensity of treatment for workers.

In addition, we found that the IMI total scores were higher in the long-training time group than the short-training time group. Because intrinsic motivation is thought to be related to training time and better performance, IMI total scores were assumed to be a significant confounder of the effect of training time on cognitive function. In fact, the association between post-treatment and baseline changes in Spotter and IMI total scores showed a significant relationship. Additional ANCOVA with IMI total scores as a covariate showed that the effect of training time on cognitive function remained significant. The effect of training time was robust even after the IMI effect was removed.

The work performance was not improved at post-treatment and follow-up compared to baseline. It even significantly worsened in the long-training group at post-treatment. This was contrary to our expectation that the long-training group should benefit more from CT than the short-training group in terms of work performance as well as cognitive function. However, since there was no significant difference in the change in work performance between the long- and short-training groups, the decline in work performance appeared to be due to external reasons other than CT.

In an attempt to explore the factors that may contribute to the change in work performance, we conducted a multiple regression analysis with potential factors including age, sex, depression tendency, IMI total scores, training time (long or short), change in cognitive subdomain scores at post-treatment from baseline. The analysis revealed that the IMI total score was a positive contributor to the change in work performance from baseline to post-treatment, and the improvement in Symbol Check was a negative contributor.

Contrary to predictions, improvements in cognitive function did not contribute to improvements in work function in a fully adjusted model. In previous studies of patients with schizophrenia, improvements in executive function led to better work performance, but accounted for only a small portion of the overall effect on work outcome (Wykes et al., 2012; Cella et al., 2017). Conversely, the present study showed that improvement in cognitive domains related to the Symbol check test contributed to a decline in work performance. Symbol check test is designed to assess working memory, executive function, and attention/concentration, which is originated from an n-back task. The exact reason why improved Symbol Check performance has a negative effect on change in work performance is not clear, but several studies have indicated its poor psychometric properties, such as relatively low correlation with a standardized one-back task and poor ability to discriminate between patients and healthy subjects (Harrison et al., 2018; Hou et al., 2020). Symbol Check requires participants to switch their attention between stimulus sequences and response options so quickly that even the healthy controls have difficulty understanding the test during the actual process.

In the present study, intrinsic motivation for CT positively contributed to improved work performance, and was also associated with improvements in attention and executive function related to Spotter test and training time. Although a causal relationship cannot be concluded, it can be inferred that subjects who were intrinsically motivated to CT had greater adherence and longer training time, leading to improved cognitive function, and that higher intrinsic motivation directly led to improved work performance. The positive contribution of intrinsic motivation to work performance is in line with previous research on schizophrenia (Nakagami et al., 2008; Gard et al., 2009; Saperstein et al., 2011; Green et al., 2012). On the other hand, unlike schizophrenia, there was no relationship between improvement in cognitive function and work performance in the workers, presumably due to relatively preserved cognitive function.

The current study had several limitations. First, we recruited participants who had previously participated in a cross-sectional cognitive assessment study, so it is possible that the sample was biased toward those with a specific interest in cognitive function. Therefore, caution should be exercised when generalizing the results. Second, this study was conducted during the spread of COVID-19, making it difficult to conduct face-to-face briefings. Instead, we sent reminder e-mails three times a week, but the importance of CT may have been better communicated in person. Third, regarding the IMI, there were missing data for one item on the Interest/Enjoyment subscale because of error (it was missing when creating the questionnaire on the web), resulting in a six-item rating for that subscale. Finally, we assessed work performance with a single visual analog scale in reference to the Stanford Presenteeism Scale, which could have been validated by including objective measures such as absenteeism (i.e., number of sick leaves).

In conclusion, our results suggest that although remote CT was not feasible enough the effects on cognitive function could be expected by increasing training time and motivation. Further research is needed, with more participants and a better training protocol to increase adherence to validate the findings.

## Data availability statement

The datasets presented in this article are not readily available because participants of this study did not agree to share their data publicly. Requests to access the datasets should be directed to MS, michis@yokohamah.johas.go.jp.

## Ethics statement

The studies involving humans were approved by the Institutional Review Board of the Japan Organization of Occupational Health and Safety. The studies were conducted in accordance with the local legislation and institutional requirements. Written informed consent for participation was not required from the participants or the participants’ legal guardians/next of kin because this was a non-interventional study in which only those who were sent the study invitation and agreed to participate in the study were included in the study. The invitation was sent so that the participants could withdraw their participation in the study at any time. A non-invasive questionnaire and cognitive function tests were used to obtain data.

## Author contributions

MS: Conceptualization, Data curation, Formal analysis, Funding acquisition, Investigation, Project administration, Writing – original draft, Writing – review & editing, Resources. MM: Data curation, Formal analysis, Writing – original draft, Writing – review & editing. SIw: Writing – original draft, Writing – review & editing, Formal analysis, Methodology, Supervision. SIk: Formal analysis, Methodology, Project administration, Supervision, Writing – original draft, Writing – review & editing. HE: Data curation, Formal analysis, Methodology, Writing – original draft, Writing – review & editing. KN: Conceptualization, Data curation, Formal analysis, Investigation, Methodology, Project administration, Supervision, Validation, Writing – original draft, Writing – review & editing.
